# Seconds Save Sight: Improving Orbital Compartment Syndrome Management Through a Closed-Loop Quality Improvement Project

**DOI:** 10.7759/cureus.115862

**Published:** 2026-09-06

**Authors:** Peter Awad, Mark Awad, John Awad

**Affiliations:** 1 East of England Foundation School, Queen Elizabeth Hospital King's Lynn NHS Foundation Trust, King's Lynn, GBR; 2 Ophthalmology, Royal Bournemouth Hospital, Bournemouth, GBR; 3 Ophthalmology, Mid Cheshire Hospitals NHS Foundation Trust, Cheshire, GBR

**Keywords:** acute orbital compartment syndrome, cantholysis, emergency preparedness, human factors, innovation in ophthalmology, lateral canthotomy, patient safety improvement, quality improvement projects

## Abstract

Introduction

Orbital compartment syndrome is a rare but sight-threatening ophthalmic emergency requiring immediate recognition and intervention to prevent irreversible vision loss. Emergency lateral canthotomy and cantholysis remain the definitive treatment; however, the infrequent nature of the procedure means many ophthalmologists have limited practical experience and reduced procedural confidence. In addition to technical familiarity, delays in treatment may arise from difficulties locating emergency equipment within clinical environments. This quality improvement project evaluated clinician preparedness for the management of orbital compartment syndrome within a UK National Health Service (NHS) ophthalmology department and assessed the impact of introducing standardised emergency lateral canthotomy grab kits alongside focused educational teaching.

Methods

A single-centre closed-loop quality improvement project was undertaken within a UK NHS ophthalmology department. Clinicians of varying grades completed a baseline questionnaire assessing confidence in recognising orbital compartment syndrome, performing emergency lateral canthotomy, and locating the equipment required to undertake the procedure under time-critical conditions. Following baseline assessment, two interventions were implemented: a focused departmental teaching session reviewing recognition and management of orbital compartment syndrome and a dedicated emergency lateral canthotomy grab kit containing all equipment required for orbital decompression. A repeat survey was undertaken following implementation to evaluate changes in clinician preparedness, procedural confidence, and perceived accessibility of emergency equipment.

Results

Baseline findings demonstrated variable confidence in performing emergency lateral canthotomy and highlighted uncertainty regarding the location of essential equipment. Following implementation of the educational intervention and emergency grab kit, clinicians reported improved preparedness and procedural confidence. Around 92% of participants agreed that the introduction of dedicated grab kits improved procedural efficiency and accessibility, while all respondents reported being able to identify the equipment required to perform emergency lateral canthotomy following implementation.

Conclusion

This quality improvement project demonstrated that simple, low-cost interventions can improve clinician preparedness for the management of orbital compartment syndrome. Combining focused education with standardised emergency equipment may reduce avoidable delays during time-critical ophthalmic emergencies and represent an easily reproducible intervention that could be adopted across ophthalmology departments nationwide to improve the management of orbital compartment syndrome.

## Introduction

Background

Orbital compartment syndrome is one of the few true ophthalmic emergencies in which irreversible vision loss may occur within a matter of hours if prompt intervention is not undertaken [1,2]. As intra-orbital pressure increases, perfusion of the optic nerve and retina is compromised; this results in ischaemic injury that may become irreversible if decompression is delayed. Although retrobulbar haemorrhage following facial trauma remains the most common cause, orbital compartment syndrome can be caused by many other pathologies [1,2]. Regardless of aetiology, timely recognition and emergency orbital decompression are fundamental to preserving vision.

Emergency lateral canthotomy with inferior cantholysis remains the definitive sight-saving procedure for orbital compartment syndrome [1-5]. The procedure rapidly decompresses the orbit by releasing the lateral canthal tendon, thereby reducing intra-orbital pressure and restoring optic nerve and retina perfusion. Visual outcomes are determined by time from diagnosis to decompression [2,5]; therefore, clinical diagnosis should prompt immediate intervention, and treatment should not be delayed while awaiting imaging or specialist review [2-4].

Available knowledge

Despite its importance, emergency lateral canthotomy is an infrequently performed procedure within routine ophthalmic practice. Consequently, many clinicians complete training having observed relatively few cases and often rely on simulation-based teaching to maintain procedural familiarity [6]. This presents a recognised challenge within healthcare, whereby clinicians are expected to perform complex, high-stakes procedures despite limited real-world exposure. Previous studies have demonstrated that confidence in performing emergency procedures declines over time without regular reinforcement, highlighting the importance of refresher education and simulation training in maintaining procedural competency [7].

Low-fidelity and low-cost simulation models have also been developed specifically for lateral canthotomy training, providing accessible methods of reinforcing procedural familiarity and clinician confidence [8,9].

Alongside technical competence, increasing attention has been directed towards the role of human factors and system-based approaches in emergency care. Delays in treatment frequently arise not solely from a lack of clinical knowledge but from organisational barriers, including inconsistent equipment storage, unclear responsibilities, interruptions and increased cognitive workload during high-pressure situations [10]. Standardising emergency equipment and reducing the number of decisions required during time-critical events has been shown to improve efficiency and reduce avoidable delays [10,11]. These principles are reflected in broader approaches to healthcare standardisation [12], while human factors-based simulation has also shown promise as a feasible approach within ophthalmology [13].

Rationale

We propose that clinician preparedness in managing orbital compartment syndrome could be enhanced by addressing two key factors: firstly, by refreshing clinical knowledge and secondly, by improving accessibility to the necessary equipment.

The first component consisted of a focused educational session reviewing the recognition, diagnosis, and emergency management of orbital compartment syndrome. The second involved the introduction of dedicated emergency lateral canthotomy grab kits containing all equipment required to perform orbital decompression. By standardising equipment and ensuring immediate accessibility, the intervention aimed to reduce cognitive load, minimise treatment delays created by equipment retrieval, and improve clinician confidence during high-pressure emergency situations. This approach reflects established principles of addressing human factors [10,11,13].

## Materials and methods

Study design

This study was conducted at a UK National Health Service (NHS) ophthalmology department (Leighton Hospital, Crewe, United Kingdom), as a single-centre closed-loop quality improvement project. A pre- and post-intervention survey design was used to evaluate clinician preparedness for the emergency management of orbital compartment syndrome before and after implementation of targeted educational teaching and standardised lateral canthotomy grab kits. The project was undertaken in accordance with the Standards for Quality Improvement Reporting Excellence (SQUIRE 2.0) guidelines [14]. The project involved anonymous questionnaires completed by clinical staff only and did not involve patient recruitment or collection of patient data.

Settings

The project was undertaken within a district general hospital ophthalmology department providing emergency and elective ophthalmic care. Ophthalmologists working within the department are expected to recognise and manage orbital compartment syndrome, including performing emergency lateral canthotomy and cantholysis when clinically indicated. Prior to this project, no dedicated emergency lateral canthotomy kits were available, and the equipment required for the procedure was stored across multiple clinical areas.

Participants

All ophthalmologists working within the department during the study period were invited to participate. A total population sampling approach was used, whereby all eligible clinicians were invited rather than selecting a sample from the department population. Inclusion criteria comprised medical staff working within the ophthalmology department who are expected, as part of their clinical role, to recognise and/or manage orbital compartment syndrome. Participants included consultant ophthalmologists, speciality doctors and ophthalmology trainees. Clinicians not working within the ophthalmology department during the study period and non-medical staff not expected to perform emergency orbital decompression were excluded from the study.

The participant group was selected because ophthalmologists within the department may be required to recognise orbital compartment syndrome and undertake emergency lateral canthotomy and cantholysis as part of their clinical role. As this was a local quality improvement project involving the entire eligible clinician population, no formal sample size calculation was performed. The target sample was therefore determined by the total number of eligible ophthalmologists working within the department (N = 17), rather than by a statistical sample size formula.

Of the 17 eligible clinicians invited to complete the baseline questionnaire, 13 participated, giving an overall response rate of 76%. The 13 clinicians comprised six consultants, five speciality doctors, one senior ophthalmology trainee and one junior ophthalmology trainee. Participation was voluntary, and all questionnaire responses were anonymised prior to analysis. All completed responses from eligible participants were included in the descriptive analysis, with no responses selectively excluded on the basis of clinician grade, previous procedural experience or confidence level.

Baseline assessment

Baseline assessment was undertaken using a purpose-designed questionnaire developed for this quality improvement project (Appendix 1). The questionnaire consisted of multiple-choice questions, five-point Likert-scale items, and free-text responses designed to evaluate clinician preparedness for recognising and managing orbital compartment syndrome.

The questionnaire collected information regarding clinician grade, previous experience performing emergency lateral canthotomy, confidence in recognising orbital compartment syndrome, self-reported confidence in performing emergency lateral canthotomy, ability to locate the equipment required for the procedure, and perceptions regarding the potential value of introducing dedicated emergency lateral canthotomy grab kits. Participants were also invited to provide free-text comments describing perceived barriers to emergency management and suggestions for service improvement.

The questionnaire was developed specifically for this quality improvement project. Self-reported confidence was measured using a five-point Likert-type scale ranging from 1 (not confident) to 5 (very confident), with higher scores representing greater perceived confidence [15].

Baseline survey findings were used to identify areas requiring improvement and inform the design of the subsequent interventions.

Intervention

Two complementary interventions were introduced following analysis of the baseline survey. The first intervention consisted of a focused departmental teaching session reviewing the recognition and emergency management of orbital compartment syndrome. Teaching included the pathophysiology of orbital compartment syndrome, recognition of key clinical signs, indications for emergency orbital decompression, relevant orbital anatomy, a stepwise demonstration of lateral canthotomy and inferior cantholysis using procedural diagrams, discussion of common procedural pitfalls and complications, equipment required to perform the procedure safely, and post-procedural management. Opportunities for questions and group discussion were provided throughout the session.

The second intervention involved the introduction of two standardised emergency lateral canthotomy grab kits, strategically located within the outpatient department and ophthalmology ward to improve equipment accessibility during time-critical emergencies. Each grab kit contained a laminated procedural reference guide, a skin marker pen, an alcohol swab, a 5 mL syringe, a 25- or 27-gauge subcutaneous needle, lidocaine with adrenaline, a haemostat clip, toothed forceps, straight cutting scissors, a disposable scalpel blade, sterile gauze and an eye pad with a protective shield. The grab kits were designed to ensure that all equipment required to perform emergency lateral canthotomy and inferior cantholysis was immediately available in a single standardised location.

Outcome measures

The primary aim of this quality improvement project was to evaluate clinician preparedness for recognising and managing orbital compartment syndrome within our department.

The secondary aims of this quality improvement project were to assess clinician confidence in performing emergency lateral canthotomy, identify barriers to accessing the equipment required for this procedure, evaluate the impact of focused educational teaching and standardised emergency lateral canthotomy grab kits on clinician preparedness/perceived procedural confidence, and determine the feasibility of implementing a low-cost and sustainable intervention that could be reproduced across other ophthalmology departments.

Data analysis

Questionnaire responses obtained before and after implementation of the intervention were analysed using descriptive statistics. Categorical variables are presented as frequencies and percentages, while Likert-scale responses are summarised using mean scores where appropriate. Pre- and post-intervention responses were compared descriptively by examining changes in response frequencies, percentages and mean Likert scores. Data were entered, organised and analysed using Microsoft Excel, version 16.109.3 (Build 26053122; Microsoft Corporation, Redmond, WA, USA). Qualitative free text responses were analysed using an inductive thematic approach to identify recurring barriers to emergency management and suggestions for future service improvement. Given the descriptive quality improvement design and small departmental population, no formal inferential statistical tests were performed; pre- and post-intervention findings were compared descriptively using frequencies, percentages, and mean Likert scores.

Ethical considerations

This project was undertaken as a local quality improvement initiative designed to improve clinical practice and service delivery within the ophthalmology department. The project involved voluntary, anonymous questionnaires completed by members of staff only; no patients were recruited, no patient data was collected, and no interventions were performed on patients. In accordance with local NHS quality improvement guidance, the project was classified as a service improvement initiative and did not require formal approval from a UK research ethics committee. The project was reviewed and authorised through the departmental quality improvement governance process prior to implementation. Completion of the questionnaire was considered to imply informed consent to participate.

## Results

Participant characteristics

Seventeen clinicians were invited to participate, of whom 13 completed the baseline questionnaire, giving an overall response rate of 76%. Participants represented a broad range of clinical experience and included six consultant ophthalmologists (46%), five speciality doctors (38%), one senior speciality trainee (8%), and one junior speciality trainee (8%) (Table 1).

**Table 1:** Participant characteristics (n = 13) Data are presented as n(%). Descriptive statistics were used; no inferential statistical testing was performed. n: number of participants.

Clinician Grade	n(%)
Consultant	6(46%)
Speciality Doctor	5(38%)
Senior Trainee	1(8%)
Junior Trainee	1(8%)
Total	13(100%)

Although the majority of respondents were senior clinicians, previous experience of performing emergency lateral canthotomy varied considerably. Four clinicians (31%) reported never having performed the procedure; seven (54%) had performed between one and three lateral canthotomies; one clinician (8%) had performed between four and six procedures; and one clinician (8%) had performed between seven and ten canthotomies (Table 2).

**Table 2:** Previous experience performing lateral canthotomy (n = 13) Data are presented as n(%). Descriptive statistics were used; no inferential statistical testing was performed. n: number of participants.

Number of Procedures Performed	n(%)
0	4(31%)
1-3	7(54%)
4-6	1(8%)
7-10	1(8%)
Total	13(100%)

Baseline questionnaire findings

Baseline questionnaire responses identified several barriers to the emergency management of orbital compartment syndrome. Although clinicians generally recognised orbital compartment syndrome as a time-critical ophthalmic emergency, self-reported confidence in independently performing emergency lateral canthotomy was variable, with a mean confidence score of 3.69 out of 5. This likely reflects the limited opportunities for practical experience encountered during routine clinical practice.

Equipment accessibility was the principal system barrier identified during the baseline assessment. Only one clinician (8%) reported being able to independently identify and locate all equipment required to perform emergency lateral canthotomy without assistance. Seven clinicians (54%) reported being unable to locate all necessary equipment, while five (38%) were unsure of the location of one or more essential instruments.

Similarly, nine clinicians (69%) anticipated that assembling all necessary equipment during an emergency would be time-consuming because instruments were stored across multiple clinical areas, while four clinicians (31%) were unsure. No respondents believed that all equipment could be assembled promptly without delay.

Clinicians also expressed strong support for the introduction of dedicated emergency lateral canthotomy grab kits. Eleven respondents (85%) strongly agreed that a standardised grab kit would improve accessibility to emergency equipment and facilitate more efficient management of orbital compartment syndrome; one respondent (8%) agreed with the proposal, while one (8%) remained neutral. No participants disagreed with the introduction of the grab kits.

Post-intervention questionnaire findings

Following implementation of the focused educational teaching session and introduction of standardised emergency lateral canthotomy grab kits, clinician preparedness for the emergency management of orbital compartment syndrome improved. Mean self-reported confidence in independently performing emergency lateral canthotomy increased from 3.69/5 at baseline to 4.75/5 following the intervention.

All participants (100%) reported being able to identify and locate the equipment required to perform emergency lateral canthotomy following the introduction of grab kits, compared with only one clinician (8%) at baseline. Furthermore, 12 of 13 clinicians (92%) agreed that the intervention improved procedural efficiency by increasing accessibility to emergency equipment.

Participants also reported increased confidence in recognising orbital compartment syndrome and undertaking emergency lateral canthotomy. Improvements were observed across clinicians of all grades, suggesting that the intervention was beneficial irrespective of previous procedural experience. Qualitative feedback indicated that clinicians valued both the focused educational teaching session and the introduction of standardised grab kits, citing improved preparedness, easier access to equipment and greater confidence in responding to this sight-threatening emergency.

Overall findings

Overall, the project identified two principal barriers to the emergency management of orbital compartment syndrome: variable clinician preparedness and poor accessibility to emergency equipment. Following implementation of the intervention, improvements were observed in self-reported procedural confidence, equipment accessibility, and clinician preparedness. We also observed overwhelmingly positive perceptions regarding the usefulness of the standardised emergency lateral canthotomy grab kits.

## Discussion

Interpretation of findings

This quality improvement project demonstrates that improving preparedness for the management of orbital compartment syndrome requires more than procedural knowledge alone. While educational interventions remain fundamental to maintaining competency in low-frequency ophthalmic emergencies, our findings suggest that organisational factors, particularly immediate access to emergency equipment, represent an equally important component of clinician preparedness.

The intervention was deliberately designed to address both individual and system-level barriers identified during the baseline assessment. Teaching reinforced recognition of orbital compartment syndrome and refreshed clinicians’ understanding of emergency lateral canthotomy. The introduction of dedicated grab kits simplified access to the equipment required to perform the procedure. Together, these interventions reduced the cognitive burden associated with managing this rare time-critical emergency, therefore allowing clinicians to focus on clinical decision-making rather than equipment retrieval.

These findings reinforce the principle that preparedness for uncommon emergencies depends upon the interaction between clinician knowledge and the environment in which the care is delivered [10,11]. Even experienced clinicians may encounter unnecessary delays when equipment is dispersed across multiple clinical areas or when there is uncertainty regarding its location. Standardising emergency equipment therefore represents a practical systems-based intervention that complements, rather than replaces, procedural education.

Comparison with existing literature

Emergency lateral canthotomy is widely recognised as a core competency for ophthalmologists, despite being performed relatively infrequently in routine practice [3,4,6]. Clinical studies have also demonstrated the effectiveness of lateral canthotomy and cantholysis in achieving orbital decompression in patients with orbital compartment syndrome [16]. Previous studies have consistently demonstrated that clinicians report reduced confidence in managing infrequent, high-stakes procedures and support the role of simulation and refresher teaching in maintaining procedural familiarity [6-9]. However, most published educational interventions focus primarily on technical skill acquisition, with comparatively little emphasis on the organisational factors that influence emergency care [10,11].

This project extends this concept by combining education with standardisation of emergency equipment. Rather than addressing procedural confidence in isolation, the intervention aims to optimise the clinical system in which emergency care is delivered. This approach recognises that improving health care processes often requires modification of the working environment alongside individual education [10,11,13].

The intervention also reflects broader principles of human factor engineering [10,11]. Standardised emergency equipment is widely used throughout healthcare, including difficult airway trolleys, major haemorrhage packs and emergency thoracotomy kits. Introducing dedicated lateral canthotomy grab kits applies the same principle to ophthalmology by reducing variation in equipment availability and minimising the cognitive workload associated with emergency equipment retrieval [12].

Clinical implications

Clinician preparedness in managing orbital compartment syndrome should be considered a systems issue rather than solely an educational challenge [10,11]. While maintaining procedural competence remains essential, departments should also ensure that clinicians can rapidly access the equipment required to undertake emergency decompression.

The intervention described in this project was inexpensive and straightforward to implement; its relatively simple design may support implementation within other ophthalmology departments. Previous studies have similarly demonstrated the feasibility of low-cost approaches to lateral canthotomy education [8,9]. Importantly, the grab kits require little additional resources because they are assembled using equipment already available within most hospitals. Adoption of similar kits across NHS ophthalmology departments may therefore represent a practical method of improving preparedness without significant financial investment.

Implementation should, however, be accompanied by ongoing education, clearly defined responsibility for checking/replenishing equipment and incorporation into departmental induction programmes. Simulation and refresher education may help clinicians maintain familiarity with an infrequently performed procedure [6-9]. Standardising equipment alone is unlikely to produce sustained improvements unless clinicians remain familiar with both the procedure and the location of the emergency equipment.

The sustainability of the intervention will therefore depend on its integration into routine departmental practice. Clearly assigned responsibility for checking and replenishing the grab kits, incorporation of their location/contents into induction for the rotating clinicians, and refresher teaching may help maintain the improvements observed following implementation. These measures are particularly important in departments with regular staff turnover, where awareness of both the procedure and emergency equipment may otherwise diminish over time.

The simplicity of the intervention also supports its transferability to other ophthalmology departments. As the grab kits were assembled predominantly from equipment already routinely available within the hospital, implementation requires limited additional infrastructure or financial investment. However, differences in staffing, existing emergency pathways and equipment availability between institutions mean that the findings of this single centre project cannot be assumed to be directly generalisable to all settings. Departments seeking to introduce a similar intervention may therefore benefit from undertaking their own baseline assessment and adapting the contents and location of the grab kits to local needs. 

Strengths and limitations

A major strength of this project is that the intervention was developed directly from barriers identified by clinicians during the baseline assessment. Rather than implementing a predetermined educational programme, the project combined targeted teaching with a systems-based intervention to address locally relevant challenges. The closed-loop quality improvement methodology also enabled evaluation following implementation; this allowed us to demonstrate the feasibility of the intervention within routine clinical practice.

Several limitations should be acknowledged. First, this was a single-centre quality improvement project involving a relatively small number of participants, which may limit the generalisability of the findings to other institutions. Second, outcomes are based on self-reported preparedness and perceived accessibility of equipment rather than objective measures of procedural performance or patient outcomes. Although improvements in preparedness are encouraging, they do not necessarily demonstrate improved technical competence or reduce treatment times during real clinical emergencies.

The rarity of orbital compartment syndrome also prevented assessment of patient-centred outcomes such as visual acuity, time to decompression, or complication rates following implementation. Finally, post-intervention evaluation was performed shortly after introducing the intervention and therefore does not determine whether improvements in preparedness are maintained over time. Continued reassessment will be important as staff rotates through the department, and new clinicians join the service.

Although objective patient outcomes could not be assessed, this reflects the inherent challenge of evaluating quality improvement initiatives for rare, time-critical emergencies such as orbital compartment syndrome. In this context, improving clinician preparedness, standardising emergency equipment and reducing systems-based barriers represent meaningful and pragmatic outcomes that may contribute to more timely emergency care and better patient outcomes.

Future directions

Future quality improvement cycles should focus on evaluating the long-term sustainability of the intervention and assessing whether periodic refresher teaching is required to maintain clinician preparedness. Incorporating simulation-based assessment and timed emergency scenarios may provide more objective measures of preparedness than self-reported confidence alone [7-9].

The intervention may also be expanded beyond ophthalmology. Patients presenting with orbital compartment syndrome are frequently first assessed by emergency medicine before ophthalmology review. Future cycles could therefore include the wider multidisciplinary team to improve emergency preparedness and strengthen collaboration across the patient pathway.

Evaluation across multiple NHS ophthalmology departments would provide valuable evidence regarding the generalisability of the intervention and help determine whether standardised emergency lateral canthotomy grab kits should become routine practice nationwide.

## Conclusions

Orbital compartment syndrome is a rare sight-threatening emergency in which clinicians' preparedness and rapid access to appropriate equipment are essential. This quality improvement project identified barriers relating to procedural confidence and equipment accessibility; it addressed these issues through focused teaching and standardised emergency lateral canthotomy grab kits. The intervention was associated with greater procedural confidence, improved preparedness and universal awareness of equipment location. These findings demonstrate the feasibility of a simple, low-cost approach combining education with equipment standardisation to improve departmental readiness for this time-critical emergency. Further quality improvement cycles should assess the sustainability of these improvements, incorporate objective measures of procedural performance and explore the transferability of the intervention to other ophthalmology departments.

## Figures and Tables

**Figure 1:**
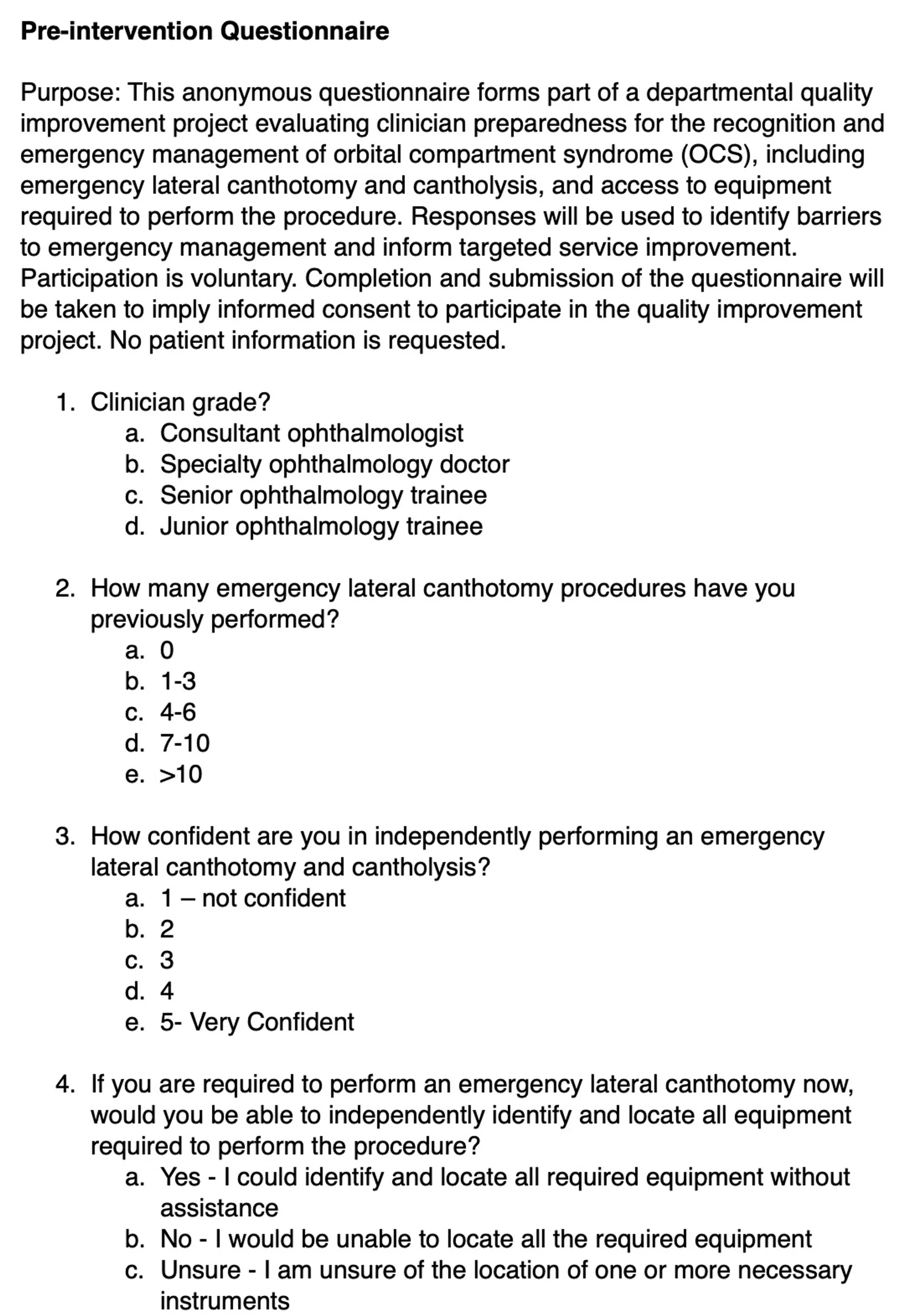
Questionnaire (part 1)

**Figure 2:**
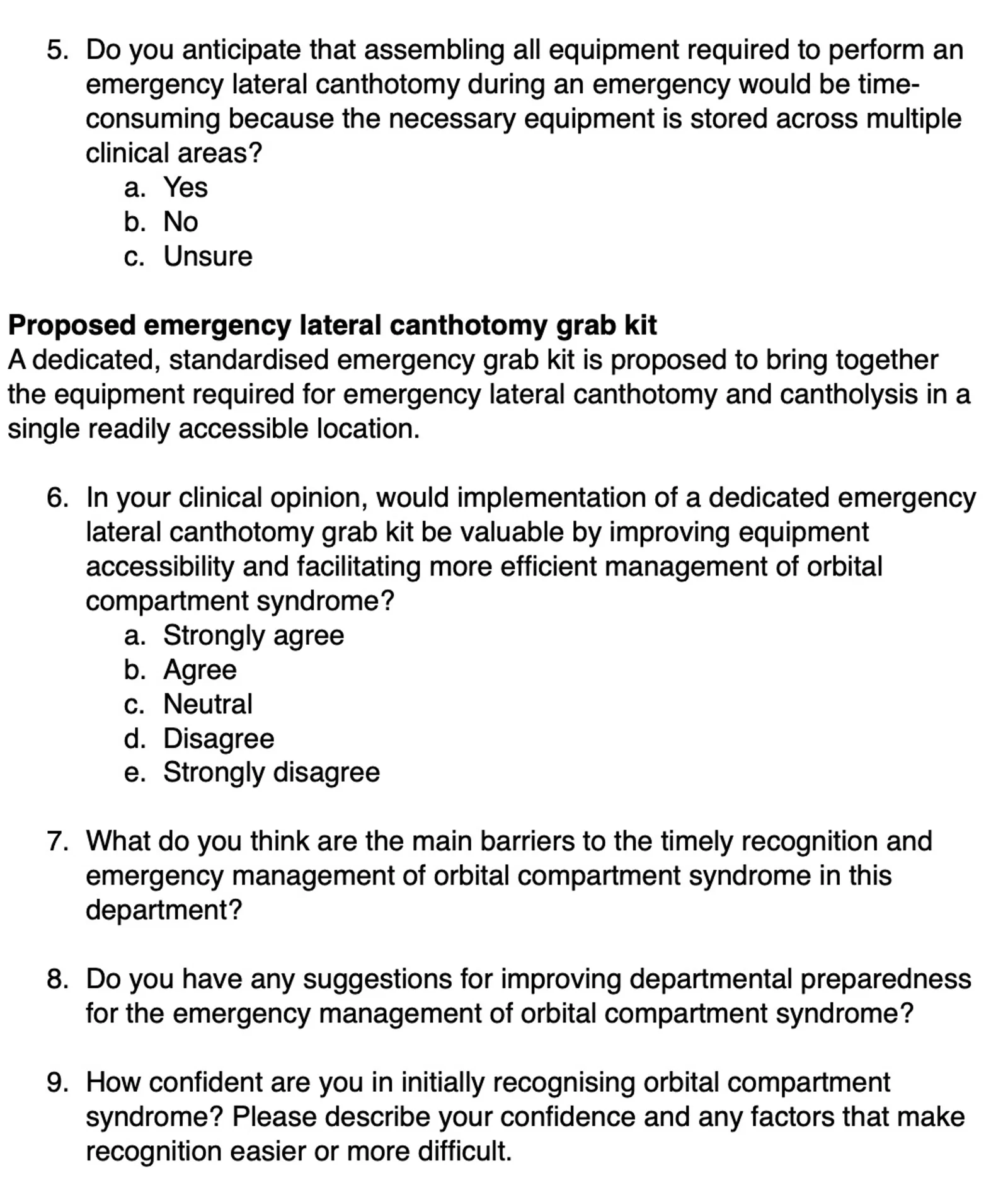
Questionnaire (part 2)

**Figure 3:**
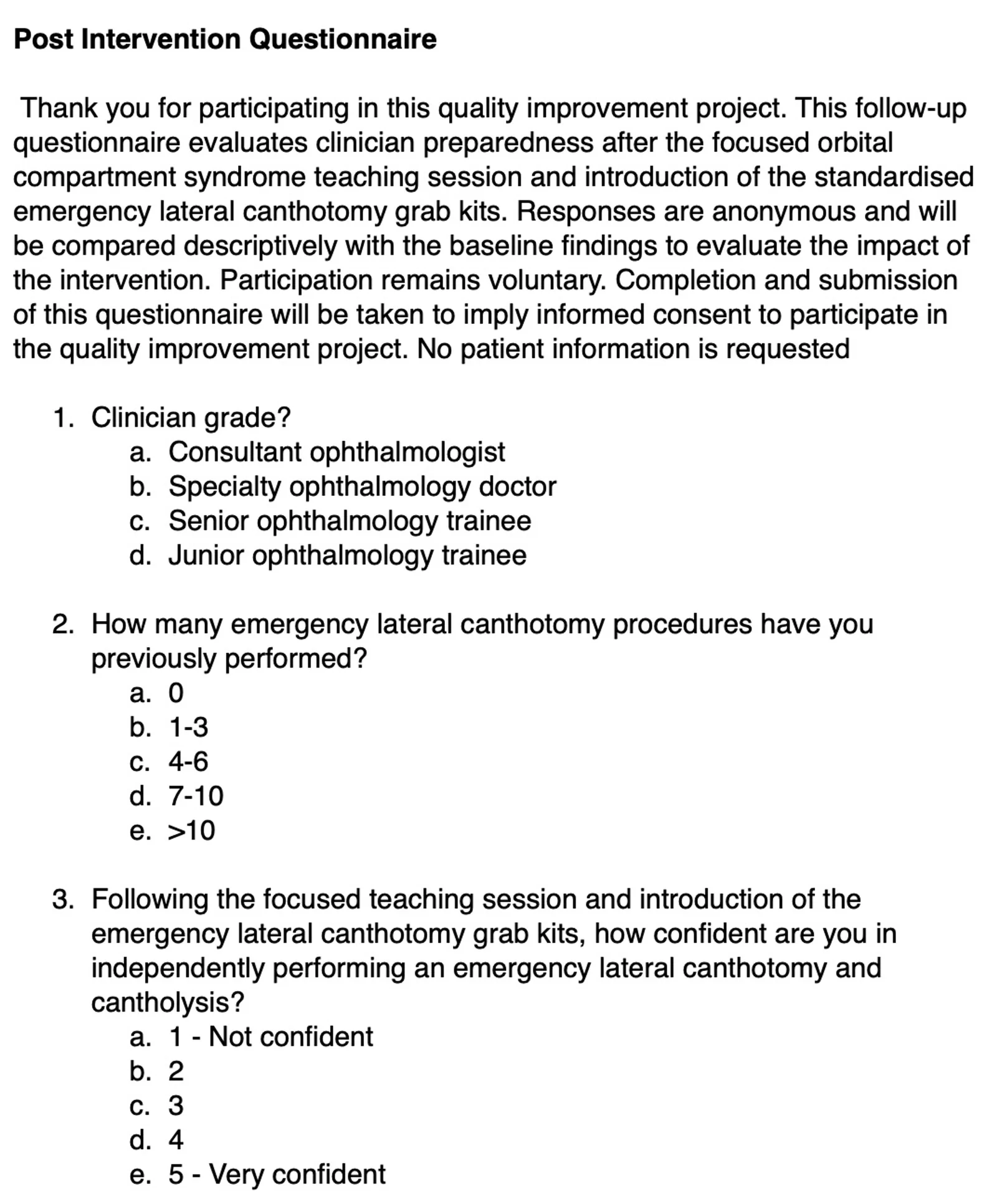
Questionnaire (part 3)

**Figure 4:**
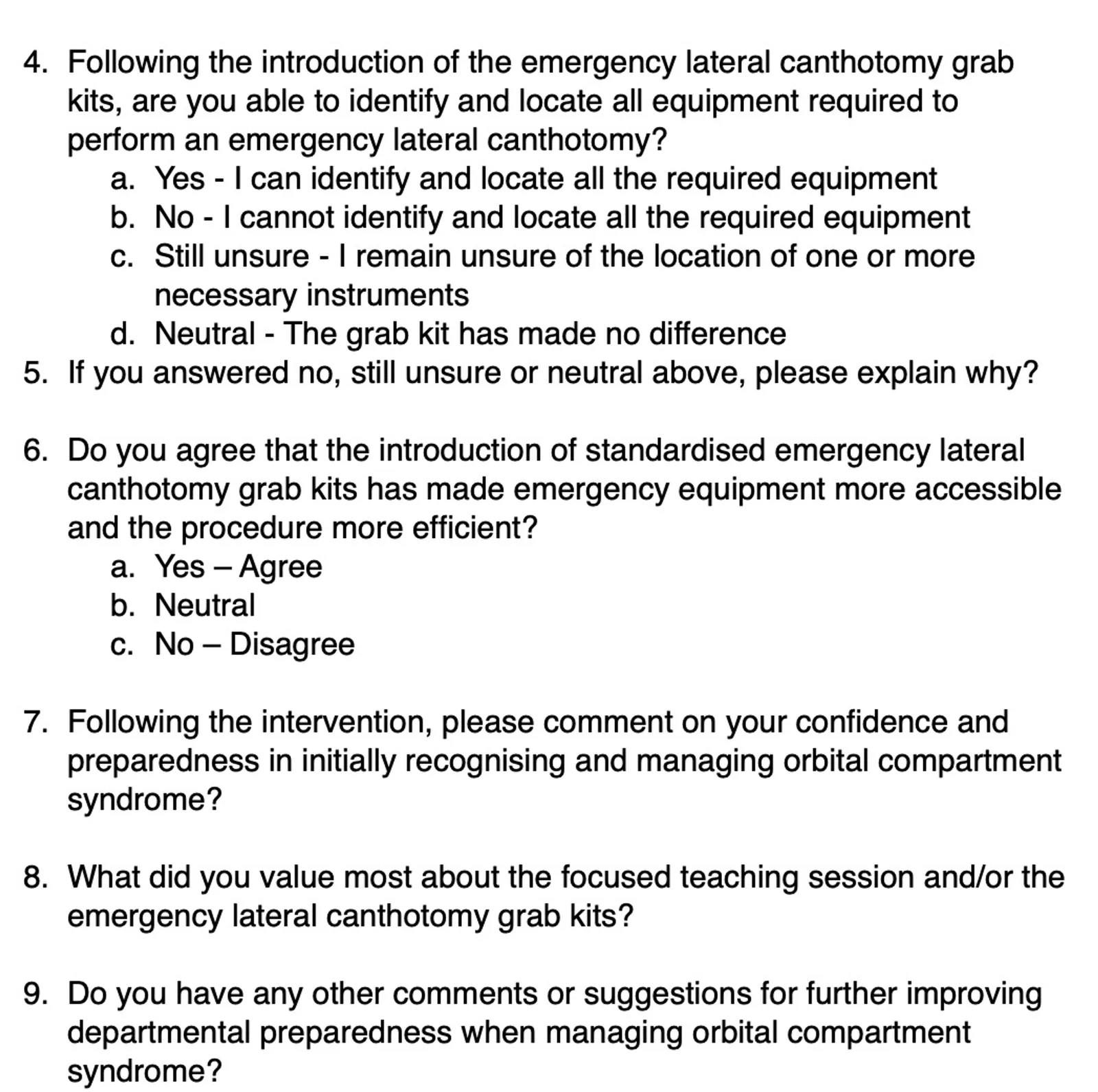
Questionnaire (part 4)

## Appendices

Appendix 1
